# Skin Aging-Dependent Activation of the PI3K Signaling Pathway via Downregulation of PTEN Increases Intracellular ROS in Human Dermal Fibroblasts

**DOI:** 10.1155/2016/6354261

**Published:** 2016-11-27

**Authors:** Eun-Mi Noh, Jinny Park, Hwa-Ryung Song, Jeong-Mi Kim, Minok Lee, Hyun-Kyung Song, On-Yu Hong, Pyoung H. Whang, Myung-Kwan Han, Kang-Beom Kwon, Jong-Suk Kim, Young-Rae Lee

**Affiliations:** ^1^Center for Metabolic Function Regulation, Wonkwang University School of Medicine, Iksan 570-749, Republic of Korea; ^2^Division of Hematology/Oncology, Gachon University Gil Medical Center, Incheon 405-760, Republic of Korea; ^3^Department of Microbiology & Immunology, Institute of Medical Science, Chonbuk National University Medical School, Jeonju 560-182, Republic of Korea; ^4^Department of Biochemistry, Institute of Medical Science, Chonbuk National University Medical School, Jeonju 560-182, Republic of Korea; ^5^Department of Pediatrics, Institute of Clinical Science, Chonbuk National University Medical School, Jeonju 560-182, Republic of Korea; ^6^Department of Korean Physiology, Wonkwang University School of Korean Medicine, Iksan 570-749, Republic of Korea; ^7^Department of Oral Biochemistry and Institute of Biomaterials, Implant, School of Dentistry, Wonkwang University, Iksan 570-749, Republic of Korea

## Abstract

Reactive oxygen species (ROS) play a major role in both chronological aging and photoaging. ROS induce skin aging through their damaging effect on cellular constituents. However, the origins of ROS have not been fully elucidated. We investigated that ROS generation of replicative senescent fibroblasts is generated by the modulation of phosphatidylinositol 3,4,5-triphosphate (PIP3) metabolism. Reduction of the PTEN protein, which dephosphorylates PIP3, was responsible for maintaining a high level of PIP3 in replicative cells and consequently mediated the activation of the phosphatidylinositol-3-OH kinase (PI3K)/Akt pathway. Increased ROS production was blocked by inhibition of PI3K or protein kinase C (PKC) or by NADPH oxidase activating in replicative senescent cells. These data indicate that the signal pathway to ROS generation in replicative aged skin cells can be stimulated by reduced PTEN level. Our results provide new insights into skin aging-associated modification of the PI3K/NADPH oxidase signaling pathway and its relationship with a skin aging-dependent increase of ROS in human dermal fibroblasts.

## 1. Introduction

Changes in the skin are the most prominent signs of aging. Skin aging can be divided into intrinsic or chronologic aging, which is the process of senescence that affects all body organs, and extrinsic aging (photoaging), which occurs because of exposure to environmental factors. One of the most important factors influencing intrinsic aging is a gradual loss of function or degeneration that occurs at the cellular level [1]. Cellular senescence, a state of essentially irreversible growth arrest of cells, can be triggered* in vitro* by phenotypic changes in morphology, gene expression, and function [2, 3]. Primary cultured cells undergo replicative senescence, which is characterized by telomere shortening, genomic damage, epigenomic damage, and activation of tumor suppressors [1].

Reactive oxygen species (ROS), primarily arising from oxidative cell metabolism, play a major role in both chronological aging and photoaging of skin [4]. Despite the presence of several antioxidative mechanisms that deteriorate with increasing age, ROS damage to cellular components still abounds. This damage leads to increasing ROS, decreasing antioxidative capacities, and finally cellular aging [4, 5]. ROS in extrinsic and intrinsic skin aging may be assumed to induce transcription factors (NF-*κ*B and c-Jun); this induction activates the decisive transcription factors, leads to the expression of matrix metalloproteinases, and prevents the expression of procollagen-1 [6]. It is still unclear what earlier events in ROS generation are involved in the progression of cellular aging. Accordingly, in this study we investigated the source of ROS in the skin cellular aging process.

The NOX family NADPH oxidases are proteins that transfer electrons across biological membranes. In general, NADPH oxidases have been thought to generate superoxide at the plasma membrane and release it into the extracellular space where it is converted into hydrogen peroxide. The biological function of NOX enzymes is the generation of ROS [7, 8]. Recent studies indicated that NADPH oxidase family members are found in a wide array of tissues [9]. NADPH oxidase is closely linked with phosphatidylinositol 3-OH kinase (PI3K) signaling [10, 11]. Protein kinase C (PKC), a downstream molecule of PI3K, is essential for superoxide generation by NADPH oxidase [10, 12].

The tumor suppressor PTEN dephosphorylates the lipid second messenger, phosphoinositol 3,4,5-trisphosphate (PIP3), an enzymatic product PI3K, and negatively regulates survival signaling mediated by PI3K/Akt (PI3K/Akt) [13, 14].

In this study, we demonstrated that PTEN downregulation and resultant activation of PI3K signaling caused PKC*ζ* activation, which in turn increased ROS production through NADPH oxidase expression and its activity modulation in replicative aged human dermal fibroblasts (HDFs). Our results provide new insights into skin aging-associated modification of the PI3K/PKC*ζ*/NADPH oxidase signaling pathway and its relationship with senescence-dependent increases of ROS in HDFs.

## 2. Materials and Methods

### 2.1. Cell Culture

HDFs isolated from neonatal foreskin were purchased from GIBCO (Invitrogen, CA). The dermal fibroblasts were cultured in Medium 106 with low serum growth supplement and 1% antibiotics at 37°C in a 5% CO2 incubator. The cells were subcultured in an atmosphere of 5% CO2 at 37°C by passaging them at a ratio of 1 : 5 in regular intervals. At later passages, the splitting ratio was reduced to 1 : 3 and 1 : 2, respectively. Cells were passaged such that the monolayers never exceeded 70–80% confluence. Population doublings (PD) were estimated using the following equation: *n* = (log 10*F* − log 10*I*)/0.301 (with *n* = population doublings, *F* = number of cells at the end of one passage, *I* = number of cells that were seeded at the beginning of one passage). The senescent status was verified by* in situ* staining for SA-*β*-galactosidase. 90–100% percent of the cells at PD 55 stained positive for SA-*β*-galactosidase.

### 2.2. Quantification of Intracellular Reactive Oxygen Species

The intracellular concentration of reactive oxygen species of HDFs was measured by using an oxidation-sensitive fluorescent probe dye, 2′,7′-dichlorodihydrofluorescein diacetate (DCF-DA) and hydroethidine (Sigma Co.) [15]. To measure intracellular ROS, the cells were incubated for 1 hr at 37°C with HBSS containing 33 *μ*M DCF-DA (Molecular Probes) or 1 *μ*M hydroethidine (Sigma Co.). The samples were then immediately observed under confocal fluorescence microscope (Olympus, Japan). The images were obtained by overlaying fluorescent images to differential interference contrast images. Also, DCF fluorescence was detected by FACStar flow cytometer (Becton Dickinson). For each sample, 10,000 events were collected. Reactive oxygen species production was expressed as mean fluorescence intensity (MFI), which was calculated by CellQuest software. Additionally, the cells were incubated for 1 hr at 37°C with HBSS containing 33 mM DCF-DA (Invitrogen Molecular Probes, Eugene, OR). The samples were then immediately observed under fluorescence microscopy.

### 2.3. Measurement of NADPH Oxidase

HDFs were lysed with the lysis buffer (20 mM Hepes, pH 7.2, 1% Triton X-100, 150 mM NaCl, 0.1 mM phenylmethylsulfonyl fluoride (PMSF), 1 mM EDTA, and 1 *μ*g/mL aprotinin). After incubation for 30 min at 4°C, cellular debris was removed by centrifugation at 10,000 ×g for 30 min. NADPH oxidase in the supernatant was measured by lucigenin chemiluminescence in the presence of 500 *μ*M NADPH (Sigma-Aldrich, St. Louis, MO) and 25 *μ*M lucigenin (Sigma-Aldrich, St. Louis, MO) as described previously [16].

### 2.4. Western Blot Analysis

Cells were lysed with ice-cold M-PER® Mammalian Protein Extraction Reagent (Pierce Biotechnology, Rockford, IL), samples (30 *μ*g) were separated by sodium dodecyl sulfate-polyacrylamide gel electrophoresis (GE Healthcare Life Sciences, Buckinghamshire, UK), and subsequent immunoblotting was performed by incubation with primary antibodies against PTEN, p85, gp91^phox^, p67^phox^, PKC*ζ*, Akt, *β*-actin (Santa Cruz, CA), phosphor-Akt (Cell Signaling Technology, Beverly, MA), and phospho-PKC*ζ* (Millipore Co., Ltd., Bedford, MA) and PIP3 (Abcam Co., MA) followed by further incubation with HRP-conjugated IgG secondary antibody. Protein expression levels were determined by signal analysis using an image analyzer (Fuji-Film, Tokyo, Japan).

### 2.5. Immunocytochemical Methods for PIP3 Analysis

Intracellular PIP_3_ levels were directly determined using an immunocytochemical method with a recently developed monoclonal antibody to PIP_3_ as described by Niswender et al. [17] Slide glasses containing HDFs were equilibrated in phosphate buffered saline at room temperature. Cells were fixed for 5–10 min at room temperature in 4% paraformaldehyde. After blocking in 5% normal goat serum and 2% bovine serum albumin, samples were incubated with mouse anti-PIP_3_ monoclonal antibody (Echelon Biosciences, UT) at a 1 : 100 dilution overnight at 4°C. The negative control for the antibody was an equivalent concentration of nonimmune mouse IgM. Primary antibodies were detected with goat anti-mouse IgM-TRITC at a 1 : 200 dilution 1 h at 4°C. Specimens were viewed using an Olympus FluoView laser scanning confocal microscope.

### 2.6. Preparation of Ectopic Expression of PTEN

To generate adenoviral constructs, the PTEN entry vector (pENTR-*PTEN*, Invitrogen, CA) and a control entry vector (pENTR-*GUS*->lacZ) were recombined with pAD/CMV/V5 (Invitrogen, Carlsbad, CA) using LR Clonase II (Invitrogen, Carlsbad, CA) to generate pAD/CMV/*GUS*->lacZ/V5 and pAD/CMV/*PTEN*/V5, respectively. The plasmid was linearized with Pac 1 and was transfected into 283A cells using lipofectamin 2000. Viruses from the culture supernatants of 293A cells that showed cytopathogenic effects were purified by cesium chloride banding. A confluent culture of HDFs was infected with recombinant adenovirus at a concentration of 1.0 plaque-forming units (pfu) per cell for 48–72 hours.

### 2.7. Statistical Analysis

Data are expressed as mean ± SEM. Statistical comparisons were performed using one-way ANOVA followed by the Fisher test. The significance of differences between groups was determined using Student's unpaired *t*-test. A value of *p* < 0.05 was accepted as an indication of statistical significance.

## 3. Results

### 3.1. Intracellular ROS Levels Are Increased by Activation of NADPH Oxidase in Replicative Aged HDFs

**Figure 1:**
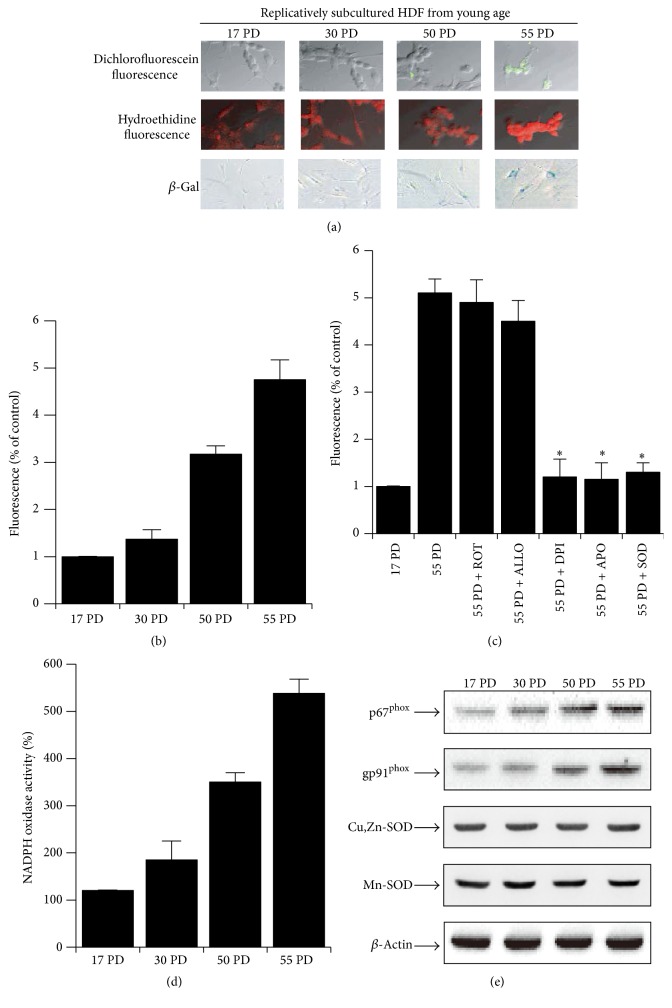
Elevation of cellular ROS levels in replicative aged HDFs through the activation of NADPH oxidase. (a) Increase in ROS with increasing PD of HDFs determined by ROS-sensitive fluorescent dyes. Various PDs of HDFs were stained with DCF-DA for 30 min and visualized with fluorescence microscopy. Senescent status was verified by* in situ* staining for SA-*β*-galactosidase. (b) ROS was measured by flow cytometry using DCFH-DA in various PDs of HDFs. (c) ROS in replicative aged HDFs (55 PD) under control conditions and in the presence of superoxide dismutase (SOD, 200 U/mL), rotenone (ROT, 10 mM), allopurinol (ALLO, 100 *μ*M), diphenyleneiodonium (DPI, 100 *μ*M), and apocynin (APO, 300 *μ*M). (d) Increase in NADPH oxidase activity with increasing PDs of HDFs. NADPH oxidase activity was measured by lucigenin activity in the presence of 500 *μ*M NADPH. (e) Multiple PDs of HDFs were analyzed by western blot for gp91^phox^, p67^phox^, and *β*-actin. Error bars, SD; *n* = 5 in each group. ^*∗*^ *P* < 0.001, compared with 55 PD.

Skin cells in the human body have limited replication potential* in vivo*. This is reflected by the finite replicative lifespan* in vitro* termed “replicative cellular senescence,” which has been proposed as an experimental model for human aging [18]. We also made an* in vitro *skin aging model by replicative subculturing with HDFs. We first analyzed whether ROS would change during extended passaging of HDFs. The level of intracellular ROS was determined using Dichlorofluorescin diacetate (DCF-DA) and Hydroethidine Fluorescence. Additionally, both of fluorescence was elevated in cells with extended HDF passages. Senescent status was verified by* in situ* staining for SA-*β*-galactosidase; 90–100% percent of the cells at PD 55 stained positive for SA-*β*-galactosidase (Figure 1(a)). Intracellular ROS levels increased during the passage progression by 4.9 ± 0.2 fold from passage 17 to 55 when ROS was quantified using a flow cytometer (Figure 1(b)). Interestingly, the increased ROS in replicative aged skin cells was not extinguished by the treatment with rotenone, an inhibitor of mitochondrial electron transfer (Figure 1(c)). However, ROS increase in replicative aged HDFs was inhibited by diphenyleneiodonium (DPI) and apocynin, inhibitors of NADPH oxidase (Figure 1(c)). Allopurinol, an inhibitor of xanthine oxidase, had no effect on aging-induced ROS (Figure 1(c)). The increased ROS in aged HDF was inhibited by superoxide dismutase (SOD) (Figure 1(c)). It is likely that the major source of ROS in replicative aged HDFs is not the mitochondria, but rather NADPH oxidase. These findings are further supported by our data that NADPH oxidase activity was elevated in replicative aged HDFs (Figure 1(d)). Additionally, we confirmed that elevated NADPH oxidase activity decreased by DPI and SOD in time-dependent manner (Supplementary Figure 1). The increase in NADPH oxidase activity can be induced by the increase of NADPH oxidase expression or modulation of NADPH activity. The expression of p67^phox^ and gp91^phox^ of NADPH oxidase increased in replicative aged HDFs. However, the expression of superoxide degrading enzymes, Cu, Zn-SOD, and Mn-SOD, did not change during the progression of cellular aging (Figure 1(e)). Together, these results indicated that increased ROS was elevated by NADPH oxidase activation in replicative aged HDFs. These results suggest that ROS increase in aged HDFs might be caused by increased NADPH expression rather than modulation of NADPH oxidase activity.

### 3.2. ROS Generation in Replicative Aged HDFs Is Induced by PI3K Signaling, Phosphorylation of PKC*ζ*, and NADPH Oxidase Activation

**Figure 2:**
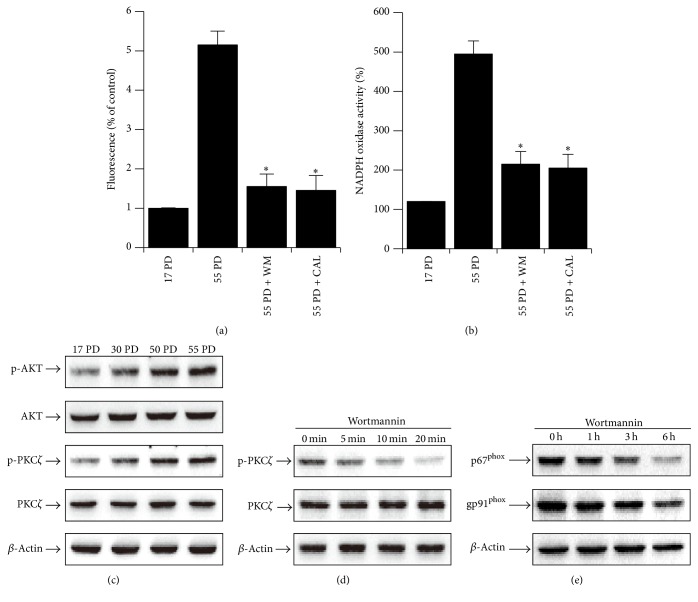
The elevation of cellular ROS levels in replicative aged HDFs through activation of PI3K, PKC*ζ*, and NADPH oxidase. (a) Blockage of aging-induced increase of ROS by incubating 55 PD HDFs with medium alone or with 100 nM wortmannin or 100 nM calphostin for 30 min. Blockage was measured by flow cytometry using DCF-DA. (b) Blockage of aging-induced increase of NADPH oxidase activity by wortmannin and calphostin C. NADPH oxidase activity was measured by lucigenin activity with medium alone or with 100 nM wortmannin or 100 nM calphostin C in the presence of 500 *μ*M NADPH. (c) Aging-induced increase of PKC*ζ* and Akt phosphorylation. Various PDs of HDFs were lysed, and western blots for phospho-Akt, phospho-PKC*ζ*, and *β*-actin were performed with the cell lysates (see Methods). (d) Inhibition of PKC*ζ* phosphorylation by wortmannin. 35 PD HDFs were treated with 100 nM wortmannin for the indicated times and the cells were analyzed with western blots for phospho-PKC*ζ* and *β*-actin. (e) Inhibition of gp91^phox^ and p67^phox^ expression by wortmannin. 55 PD HDFs were treated with 100 nM wortmannin for the indicated times. The cells were analyzed by western blotting for gp91^phox^, p67^phox^, and *β*-actin. Error bars, SD; *n* = 5 in each group. ^*∗*^ *P* < 0.005, compared with 55 PD.

In order to understand the signaling pathways via ROS generation, we tested whether wortmannin and calphostin, inhibitors of PI3K and PKC, respectively, inhibited the enhanced NADPH oxidase activity and ROS levels in replicative aged HDFs (Figures 2(a) and 2(b)). These findings suggest that ROS generation through the activation of NADPH oxidase in replicative aged HDFs might be mediated by PI3K and PKC signaling. From this finding we expected that PI3K and PKC signaling could be activated in replicative aged HDFs. Indeed, the phosphorylation of Akt, a hallmark of PI3K signal activation, increased in replicative aged HDFs (Figure 2(c)). PKC*ζ* phosphorylation, an indicator of PKC activation, was also elevated in replicative aged HDFs (Figure 2(c)). PKC*ζ* phosphorylation is likely to be mediated by activation of the PI3K-Akt pathway because PKC*ζ* phosphorylation was inhibited by treatment with wortmannin (Figure 2(d)). Also, the expression of p67^phox^ and gp91^phox^ was decreased by 6 h treatment with wortmannin (Figure 2(e)). Thus, we conclude that the activation of the PI3K-Akt pathway induces phosphorylation of PKC*ζ*, leading to enhanced NADPH oxidase activity and resulting in an increase in ROS generation.

### 3.3. Replicative Aged HDFs Exhibit Increased Intracellular PIP3 Levels through PTEN Downregulation

**Figure 3:**
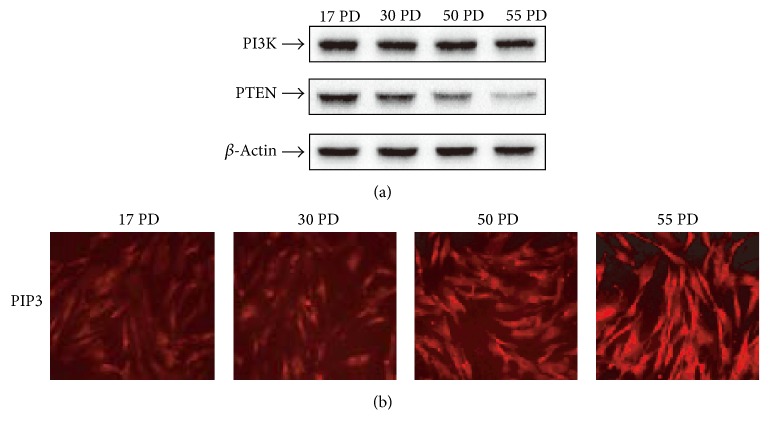
Replicative senescence-dependent increase in intracellular PIP_3_ levels through PTEN downregulation. (a) Expression levels of PI3K and PTEN, in various PDs of HDFs. Various PDs of HDFs were analyzed by western blotting for PTEN, PI3K p85 subunit, and *β*-actin. (b) PIP_3 _immunofluorescence staining in various PDs of HDFs. Various PDs of HDFs were fixed with 4% paraformaldehyde, incubated with anti-PIP_3 _antibody and stained with TRITC-labeled anti-mouse IgM antibody. Images were acquired using an Olympus FluoView™ laser scanning confocal microscope.

To determine what activates the PI3K-Akt pathway, we tested whether the protein levels of PI3K increase as HDFs age. We determined that the PI3K p85 subunit protein levels showed a slight decrease in replicative aged HDFs (Figure 3(a)). PI3K signaling can be affected by PTEN since the PIP3 level is controlled directly by the balance of activities between PI3K, the synthetic enzyme of PIP3, and PTEN, its degradative enzyme [18, 19]. We found that the protein level of PTEN was reduced much more than that of PI3K as the cells aged (Figure 3(a)). These data suggest that the imbalance between PI3K and PTEN levels induces modulation of PIP3 metabolism, which results in the activation of PI3K-Akt pathway. To confirm this, we measured intracellular PIP3 levels in HDFs of various ages. Intracellular levels of PIP3 increased with increasing passages of HDF indicating that the elevation of PIP3 levels in replicative aged HDFs is induced by a decrease in PIP3 breakdown through greater downregulation of PTEN than of PI3K (Figure 3(b)). Additionally, we confirmed elevation of PIP3 protein expression with increasing passage of HDF using western blotting analysis (Supplementary Figure 2). And we examined the effect of PI3K on PIP3 production. Treatment of Wortmannin (PI3K inhibitor) decreased PIP3 expression in aged cells (Supplementary Figure 3).

### 3.4. Overexpression of PTEN Decreases Cellular ROS Levels by Inhibiting NADPH Oxidase through PIP3 Downregulation

**Figure 4:**
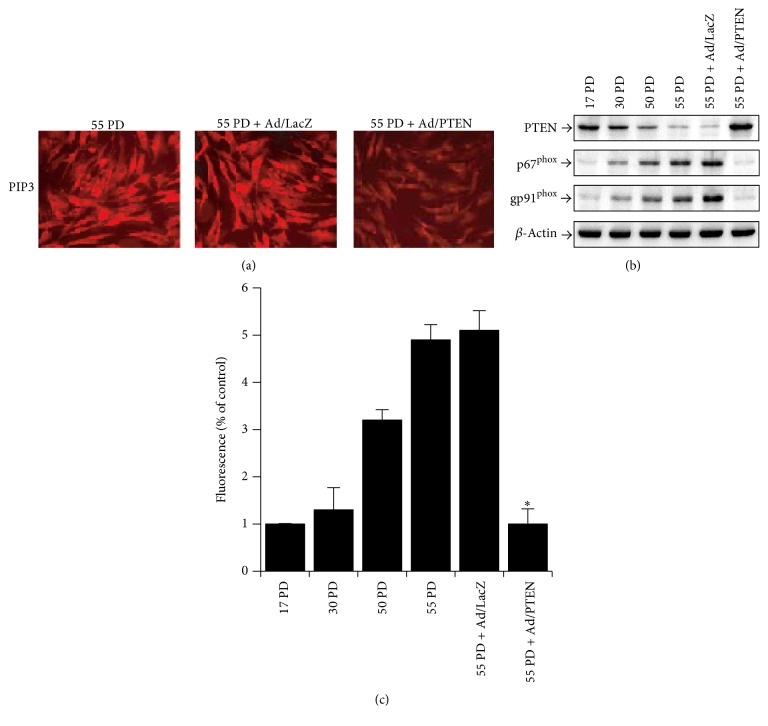
ROS generation in replicative aged HDFs is induced by the activation of PI3K signaling through aging-induced downregulation of PTEN and activation of PI3K/PKC. (a) The inhibition of aging-induced increases in PIP3 levels by PTEN gene transfer. Replicative aged HDFs (55 PD) were cultured in standard medium and infected with Ad/LacZ and Ad/PTEN. After 48 h, the cells were fixed with 4% paraformaldehyde, incubated with anti-PIP3 antibody, and stained with TRITC-labeled anti-mouse IgM antibody. Images were acquired with an Axiovert S100 fluorescence microscope (Zeiss, Germany) equipped with a DP70 digital camera (Olympus, Japan). (b) Inhibition of p91^phox^ and p67^phox^ expression by PTEN gene transfer. Replicative aged HDFs (55 PD) were cultured in standard medium and infected with Ad/LacZ or Ad/PTEN. After 48 h, the cells were lysed and blotted with antibody for PTEN, gp91^phox^, p67^phox^, and *β*-actin. (c) Inhibition of ROS generation by PTEN gene transfer. Replicative aged HDFs (55 PD) were cultured under standard medium and infected with vector alone (Ad/LacZ) or PTEN viral vector (Ad/PTEN). After 48 h, ROS was measured by flow cytometry using DCF-DA. Error bars, SD; *n* = 5 in each group. ^*∗*^ *P* < 0.005, compared with 55 PD.

To confirm the effect of PTEN on NADPH oxidase/ROS, we infected replicative aged HDFs with adenovirus containing the PTEN gene (Ad/PTEN) or control lacZ (Ad/LacZ). PTEN protein levels were elevated by infection with Ad/PTEN (Figure 4(b)). PTEN overexpression in replicative aged HDFs abolished the aging-induced increases in PIP3 concentration (Figure 4(a)) and NADPH oxidase expression (Figure 4(b)) as well as the resultant aging-induced ROS generation (Figure 4(c)). On the contrary, inactivation of PTEN increased PIP3 expression (Supplementary Figure 4). Our data show that enhanced ROS further activates PI3K signaling by the inactivation of PTEN.

## 4. Discussion

ROS induces skin aging by damaging cellular constituents; however, the origins of ROS have not been elucidated in intrinsic skin aging. In this study, we showed that ROS in replicative aged HDFs are generated by the modulation of PIP3 metabolism. The decrease in PTEN protein, which dephosphorylates PIP3, was responsible for maintaining high levels of PIP3 in replicative aged HDFs and consequently mediated the activation of the PI3K/Akt pathway. PI3K/Akt pathway activation led to PKC*ζ* activation and in turn increased ROS production through increases in NADPH expression. Therefore, we investigated whether the pathway leading to ROS generation is initiated by increased PIP3 level through regulation of PTEN in replicative aged HDFs.

Skin aging, a progressive and multifactorial but not yet fully understood process, is particularly interesting because of the continuously increasing life expectancy in many countries [20, 21]. Changes in the skin are the most prominent signs of aging. Skin aging can be divided into intrinsic or chronologic aging, which is the process of senescence that affects all body organs, and extrinsic aging caused by environmental factors. Intrinsic or natural mechanisms play a role in the way an individual ages, and both intrinsic and extrinsic mechanisms share molecular pathways. In this study, we investigated the signal pathway of ROS generation with a focus on the mechanisms of skin aging.

ROS, byproducts of normal cellular oxidative processes, increase as cellular senescence progresses [22, 23] and have been shown to contribute to cellular senescence [24]. ROS primarily arise from oxidative cell metabolism and play a major role in both chronological aging and photoaging [20]. However, it remains unclear what earlier events lead to increased ROS during the skin aging. Skin cells in the human body have a limited replication potential* in vivo*. This is reflected by the finite replicative lifespan* in vitro* termed “replicative cellular senescence,” which has been proposed as an experimental model for human aging [18]. We created an* in vitro *skin aging model by replicative subculture of HDFs. We analyzed whether ROS would change during extended passaging of HDFs (Figure 1). Interestingly, the increased ROS in replicative aged HDFs was not extinguished by treatment with rotenone, an inhibitor of mitochondrial electron transfer. The ROS increase in replicative aged skin cells was inhibited by diphenyleneiodonium and apocynin, inhibitors of NADPH oxidase. Allopurinol, an inhibitor of xanthine oxidase also had no effect on aging-induced ROS. It is likely that the major source of ROS in replicative aged skin cells is not mitochondria but NADPH oxidase. These findings are further supported by data suggesting that NADPH oxidase activity was elevated in replicative aged HDFs.

NADPH oxidases (NOX) have been thought to generate superoxide at the plasma membrane and release it into the extracellular space where it is converted into hydrogen peroxide. The biological function of NOX enzymes is the generation of ROS [7, 8]. Recent studies indicate that NADPH oxidase family members are found in a wide array of tissues [9]. ROS produced by the NOX proteins Nox1–5 and Duox1/2 play essential roles in the physiology of the brain, the immune system, the vasculature, and the digestive tract as well as in hormone synthesis. In particular, NADPH oxidase-2 is a key regulator of human dermal fibroblasts [25]. We confirmed that expression of NOXs in aged HDFs was increased (Supplementary Figure 5). NADPH oxidase is a multicomponent enzyme; the classical phagocytic NADPH oxidase is composed of membrane-bound subunits p22^phox^ and gp91^phox^ (also referred to as NOX2) as well as cytosolic subunits, including p67^phox^ and p47^phox^ [8, 26]. In our results, we confirmed that the expression of p67^phox^ and gp91^phox^ increased during the extended passaging of HDFs (Figure 1). These results indicate that the elevation of ROS levels in replicative aged HDFs is induced by an increase in NADPH oxidase-2 protein expression.

PKC*ζ* was originally discovered as a unique PKC isotype. It is classified into the atypical PKC (aPKC) subfamily [27]. PKC*ζ* is also activated by lipid components, such as phosphatidylinositols (PIs), phosphatidic acid, arachidonic acid, and ceramide. Among these lipids, PIP3 has been the focus of much interest with regard to its regulation of aPKCs in various cells. aPKCs can be regulated by PI3K, which produces PIP3 from PIP2 [28].

NADPH oxidase is closely linked with PI3K signaling [10, 11]. PKC*ζ*, a downstream molecule of PI3K, is essential for superoxide generation by NADPH oxidase [10, 12]. Thus, we tested the effect of PI3K and PKC inhibitors on aging-induced increase of ROS generation, NADPH oxidase activity and protein expression (Figure 2). In addition, we confirmed that PKC inhibitor inhibits NADPH oxidase activity in a time-dependent manner in replicative aged HDFs (Supplementary Figure 6). From our results, we conclude that activation of the PI3K/Akt pathway induces phosphorylation of PKC*ζ*, leads to enhance of NADPH oxidase expression and activity, and thereby results in increased ROS generation.

To determine what activates the PI3K-Akt pathway, we first tested whether the protein levels of PI3K increased as HDFs aged. Contrary to our expectations, the protein levels of the PI3K p85 subunit showed a slight decrease in replicative aged HDFs (Figure 3(a)). PI3K signaling can be affected by PTEN since PIP3 levels are controlled directly by a balance of activities between the PI3K, the synthetic enzyme of PIP3, and PTEN, its degradative enzyme [18, 19]. We found that, as the cells aged, the protein levels of PTEN were much more reduced than those of PI3K. These data suggest that the imbalance between PI3K and PTEN levels induces modulation of PIP3 metabolism, which results in the activation of the PI3K-Akt pathway. To confirm this, we measured intracellular PIP3 levels in HDFs of various ages and found that intracellular levels of PIP3 increased with increasing passage of HDFs. These results indicate that the elevation of PIP3 levels in replicative aged HDFs is induced by a decrease in PIP3 breakdown through greater downregulation of PTEN than of PI3K.

PTEN downregulation might be an initiation step of the signal pathway leading to ROS production. To confirm this, we examined whether the signal pathway could be stopped by restoring PTEN levels in replicative aged HDFs to the level of young HDFs. We infected replicative aged HDFs with adenovirus containing the PTEN gene (Ad/PTEN) or adenovirus containing the lacZ gene (Ad/LacZ). PTEN overexpression in replicative aged HDFs by the PTEN gene abolished the aging-induced increase in PIP3 concentration, NADPH oxidase-2 related protein expression, and consequent aging-induced ROS generation (Figure 4). These data indicate that the signal pathway for ROS generation in replicative aged HDFs can be stimulated by reduced PTEN levels. Taking that report and our results together, enhanced ROS production might further activate the PI3K/Akt pathway by PTEN inactivation, thus establishing a self-perpetuating cycle leading to further aging.

In conclusion, we demonstrated that PTEN downregulation and resultant activation of PI3K signaling caused PKC activation, which in turn increased ROS production through NADPH oxidase protein expression and activity modulation. Thus, our results provide novel evidence that ROS in replicative aged HDFs might be produced by aging-induced modifications of related cellular signal pathways. We also demonstrated that PTEN downregulation initiates the signal pathway leading to ROS generation in the progress of replicative skin aging.

## Supplementary Material

Supplementary figure 1: NADPH oxidase activity increases in aged HDFs.
Supplementary figure 2: The expression of PIP3 increases with increasing PD of HDFs.
Supplementary figure 3: Increased PIP3 levels inhibited through suppression of PI3K in aged HDFs.
Supplementary figure 4: Inactivation of PTEN increases PIP3 expression.
Supplementary figure 5: Expression of NOXs in young and aged HDFs.
Supplementary figure 6: Elevation of NADPH oxidase activity decreased by PKCζ inhibition in aged HDFs.
