# Supplementary material for: Skin Aging-Dependent Activation of the PI3K Signaling Pathway via Downregulation of PTEN Increases Intracellular ROS in Human Dermal Fibroblasts
Source: Oxid Med Cell Longev. 2016 Nov 27;2016:6354261. doi: 10.1155/2016/6354261 (PMC5149682; doi:10.1155/2016/6354261)

Supplementary figure legends

Supplementary figure 1: NADPH oxidase activity increases in aged HDFs.
Cells were treated with SOD (200 unit/ml) and DPI (5 µM) for 1 h and then collected. Cell lysates were diluted with NDAPH oxidase assay buffer and analyzed for every 1 min for 15 min by luminometer. Data are the mean ± SEM of three independent experiments.

Supplementary figure 2: The expression of PIP3 increases with increasing PD of HDFs.
The expression of PIP3 is determined by Western blotting. β-actin was used for loading control.

Supplementary figure 3: Increased PIP3 levels inhibited through suppression of PI3K in aged HDFs.
Cells were treated with 1 µM WM (wortmannin) for 1 h and then determined to PIP3 level. Cells were fixed and then incubated with anti-PIP3 antibody. Images and relative fluorescence intensity were acquired using an Olympus FluoView™ laser scanning confocal microscope. Data are expressed as the means from triplicate determinations (mean ± SEM).

Supplementary figure 4: Inactivation of PTEN increases PIP3 expression.
Cells (17 PD) were transfected with mutant (mt) PTEN plasmid by lipofectamin 3000. After transfection 24 h, cells were harvested, analyzed by Western blotting for PIP3, PTEN and β-actin.

Supplementary figure 5: Expression of NOXs in young and aged HDFs.
RNA was isolate from young (PD 17) and aged (PD 55) HDFs. The expression of NOXs was measured by real-time PCR. 18s was used as normalized gene and expression of mRNA levels was described as fold change. Data are expressed as the mean ± SEM of three independent experiments.

Supplementary figure 6: Elevation of NADPH oxidase activity decreased by PKCζ inhibition in aged HDFs.
PKCζ inhibitor (5 µM) treated for 1 h in aged HDFs. NADPH oxidase activity was determined by photoemission (RLU) every 1 min. Data are the mean ± SEM of three independent experiments.

Supplementary Figure 1

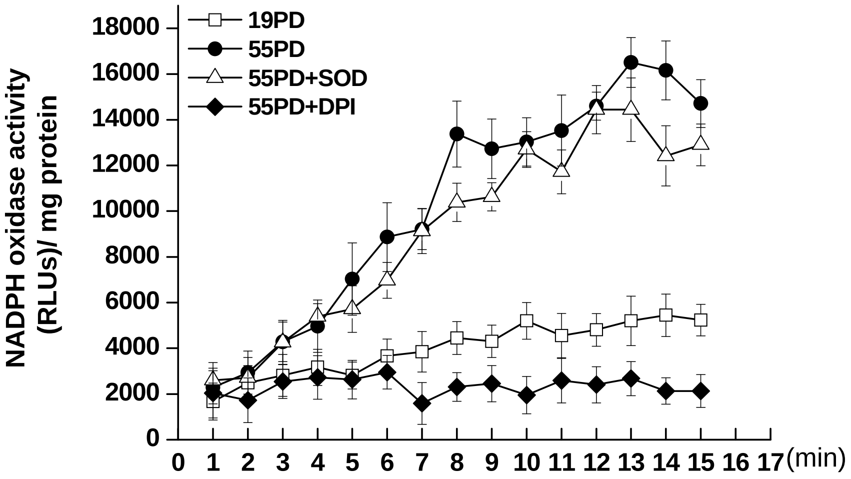

Supplementary Figure 2

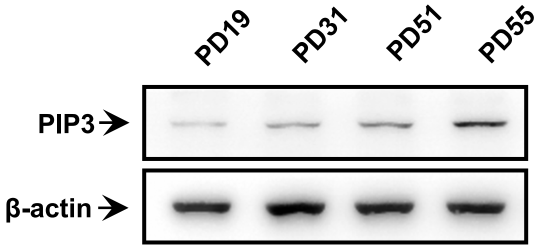

Supplementary Figure 3

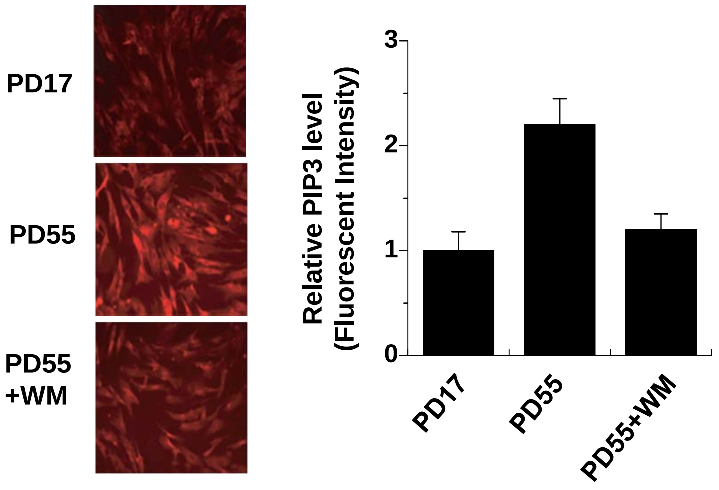

Supplementary Figure 4

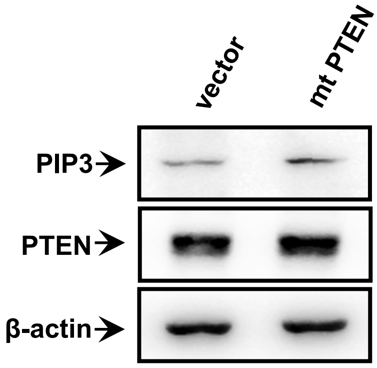

Supplementary Figure 5

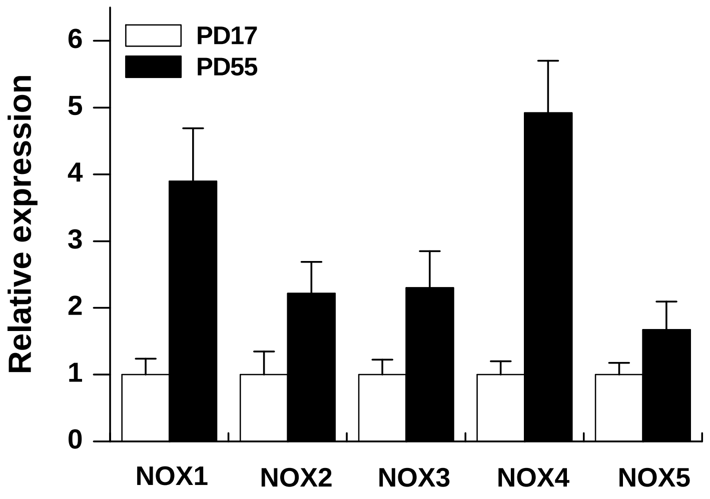

Supplementary Figure 6

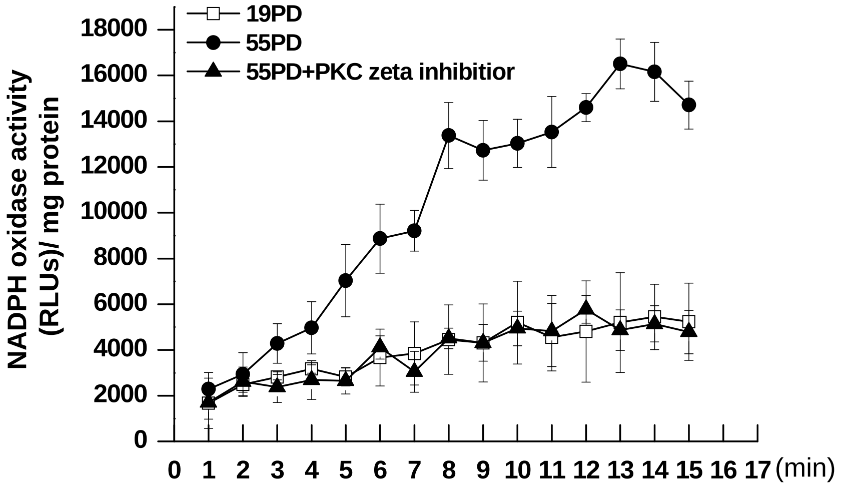

Supplement: Supplementary file 1 — Supplementary figure 1: NADPH oxidase activity increases in aged HDFs. Supplementary figure 2: The expression of PIP3 increases with increasing PD of HDFs. Supplementary figure 3: Increased PIP3 levels inhibited through suppression of PI3K in aged HDFs. Supplementary figure 4: Inactivation of PTEN increases PIP3 expression. Supplementary figure 5: Expression of NOXs in young and aged HDFs. Supplementary figure 6: Elevation of NADPH oxidase activity decreased by PKCζ inhibition in aged HDFs. [file 6354261.f1.pdf]
